# Xianfanghuomingyin, a Chinese Compound Medicine, Modulates the Proliferation and Differentiation of T Lymphocyte in a Collagen-Induced Arthritis Mouse Model

**DOI:** 10.1155/2016/6356871

**Published:** 2016-08-30

**Authors:** Bo Nie, Xue Li, Yi Wei, Meng Chen, Jingwei Zhou, Lixia Lou, Bin Dong, Aiming Wu, Dongmei Zhang, Lingqun Zhu, Jiuli Zhao, Limin Chai

**Affiliations:** ^1^Key Laboratory of Chinese Internal Medicine of Ministry of Education and Beijing, Dongzhimen Hospital, Beijing University of Chinese Medicine, Beijing, China; ^2^School of Preclinical Medicine, Beijing University of Chinese Medicine, Beijing, China; ^3^Department of Rheumatology, Dongzhimen Hospital, Beijing University of Chinese Medicine, Beijing, China

## Abstract

In traditional Chinese medicine (TCM), xianfanghuomingyin (XFHM) is used to treat autoimmune diseases, including rheumatoid arthritis (RA). Here, we studied the mechanisms underlying its treatment effects, especially its anti-inflammatory effects in a collagen-induced arthritis (CIA) mouse model. We found that cartilage destruction and pannus formation were alleviated by treatment with XFHM. The abnormal differentiation of Th1 and Th17 cells was downregulated significantly by XFHM, and Th2 and Treg cells were upregulated. Moreover, the expression levels of specific cytokines and transcription factors related to Th1 cells (interferon *γ* [IFN*γ*], T-bet) and Th17 cells (interleukin- [IL-] 17) and the nuclear receptor retinoic acid receptor-related orphan receptor-gamma (ROR*γ*) were downregulated. Serum IL-4 and GATA-3, which contribute to Th2 cells differentiation, increased significantly after XFHM administration. These results indicate that XFHM can restore the balance of T lymphocytes and reestablish the immunological tolerance to inhibit autoinflammatory disorder of RA. Taken together, XFHM can be used as a complementary or alternative traditional medicine to treat RA.

## 1. Introduction 

Rheumatoid arthritis (RA), characterized by inflammation of synovial joints, is a chronic and systemic autoimmune disease, which leads to a progressive destruction of articular cartilage and periarticular structures [1, 2]. The pathogenesis of RA is not well understood. Various inflammatory cells, including T and B cells, macrophages, dendritic cells and natural killer cells, infiltrate RA joints and induce the proliferation of cells of the synovial lining [3, 4]. These cells constitute the synovial lining, participate in inflammation, contribute to synovial proliferation, pannus formation and cartilage destruction and subchondral bone erosion [5].

The involvement of autoreactive T cells in the pathogenesis of RA well established. T helper 1 (Th1) and Th17 cell subsets play key roles in the pathogenesis of RA. Abnormal differentiation of Th1 cells, with an increased secretion of interferon *γ* (IFN*γ*) and a disturbed balance of Th1-Th2 cells, is also shown in autoimmune diseases, including RA [6, 7]. In recent years, the discovery of IL-17-producing Th17 cells has changed the initial recognition of Th1 cell driven disease of RA. Th17 cells and IL-17 trigger synovial inflammation, contributing to cartilage and bone destruction [8, 9]. Collagen-induced arthritis (CIA) is suppressed significantly in IL-17-deficient mice, suggesting that Th17 cells play a crucial role in the pathogenesis of CIA mice [10]. Regulatory T (Treg) cells that have lost regulatory control of effector T cells also promotes the pathogenesis of RA [7]. Loss of the suppression effect of Treg cells on effector T cells can lead to loss of control of the autoimmune inflammation in RA and subsequent pathological damage process. Transferring CD4^+^CD25^+^ Treg cells into CIA mice can slow the progression of CIA and reduce the production of acute-phase proteins [11].

Xianfanghuomingyin (XFHM), described in ancient herbal formulae (*JiaozhuFurenLiangfang*, which is a Chinese medical treatise compiled by XueJi in the Song Dynasty of Ancient China), is a typical traditional Chinese medicine (TCM) formula which has been used for over 1,000 years. The formulae combined plant species and minerals that were prescribed based on clinical experience. Multiple components of the formula could interact with multiple targets and exert synergistic therapeutic efficacies [12, 13]. The optimized formula of XFHM (once named Lijie capsule) used in this study is comprised of 12 different herb. Resent evidence indicates that XFHM can be used to treat osteoarthritis of the knee [14] and hip joint synovitis [15]. Additionally, our previous studies have suggested that XFHM may induce the apoptosis in lymphocytes in the peripheral blood (PB) of rats with adjuvant arthritis [16].

Here, we investigated the possible anti-inflammatory mechanisms underlying the regulation of T lymphocyte differentiation to maintain immunological tolerance following XFHM treatment in a CIA mouse model. Leflunomide (LEF), a disease-modifying antirheumatic drug (DMARD) for the management of RA [17], was used as the positive control. The aim of the present study was to identify the specific cellular targets involved in the antiautoimmune effects of XFHM.

## 2. Materials and Methods

### 2.1. Drugs

The XFHM, formula comprises* Atractylodes lancea*,* Ligusticum chuanxiong *Hort,* Paeonia veitchii *Lynch,* Tail of Radix Angelicae sinensis*,* Angelica dahurica*,* Radix Saposhnikoviae*,* Boswellia carteri *Birdw,* Commiphora myrrha*,* Astragalus membranaceus*,* Caulis Lonicerae Japonicae*,* Gentiana macrophylla *Pall, and* Rehmannia glutinosa* Libosch at a ratio of 2 : 2 : 2 : 2 : 3 : 3 : 1 : 1 : 8 : 6 : 3 : 3. Quality control was carried out by infrared fingerprint spectrum (IRFP) techniques. The IRFP graph is shown in S1 Fig. (see Supplementary Material available online at http://dx.doi.org/10.1155/2016/6356871).

### 2.2. High Performance Liquid Chromatography-Electrospray Ionization/Mass Spectrometer (HPLC-ESI/MS^n^) Analysis

HPLC-ESI/MS^n^ analysis was performed on a Shimadzu 20LC (Kyoto, Japan) coupled to a diode array detector and TripleTOF 4600 + CDS mass spectrometer (AB Sciex, MA, USA). The chromatographic separations were carried out on a Agilent Poroshell C18 (2.1 mm × 100 mm 2.7 *μ*m). The mobile phase consisted of a combination of A (0.5‰ formic acid and 2 mM acetic acid) and B (0.5‰ formic acid and 2 mM acetic acid in acetonitrile methyl alcohol [1 : 1]) with a linear gradient, 0–−10 min (5–−20%, B) or 10–−22 min (20–−95%, B). The flow rate was 0.4 mL/min, the sample injection volume was 5 *μ*L, and the column and sample temperature were 40°C. The diode array detector (DAD) was set at 200, 220, 250, and 280 nm for real-time monitoring of the peak intensity. Mass spectra were simultaneously acquired using electrospray ionization with positive and negative ionization (POS and NEG) modes at fragmentation voltages (40 psi) over the range of *m*/*z* 50–1250.

### 2.3. Mice

DBA1/J male mice (*n* = 6 per group, 7-8 weeks old) were purchased from HFK Bioscience Co. Ltd. (Beijing, China). Animal care and use were in accordance with institutional guidelines and all animal experiments were approved by the Institutional Animal Care and Use Committee of the National Institute of State Scientific and Technological Commission.

### 2.4. Induction of CIA

Mice, except mice in the normal group, were immunized intradermally at the base of the tail with 150 *μ*g of bovine type II collagen (CII) (Sigma, CA, USA) emulsified with an equal volume of complete Freund's adjuvant (CFA) (Sigma, CA, USA). Mice were boosted by intradermal injection with 150 *μ*g of CII emulsified with incomplete Freund's adjuvant (IFA) on the 21st day after immunization.

### 2.5. Drug Treatment

Drug treatment began after booster immunization and lasted for 28 days. Mice were randomly divided into four groups as follows: normal group, mice fed the control diet and orally given sterile saline; model group, mice fed the same as the normal group; LEF group (Batch number 130126, Cinkate Corporation, Beijing, China), mice fed the control diet and orally daily given 2 mg/kg LEF for 28 days; XFHM group, mice fed the control diet and orally given 5.3 g/kg XFHM daily for 28 days. The mice were sacrificed on the 29th day after treatment. Mice were anesthetized by isoflurane anesthesia (2-3% isoflurane with oxygen supply). PB was obtained by removing eyeballs, and the left legs and hind paws and spleens were removed.

### 2.6. Clinical Scores

Clinical CIA scores were monitored every 7 days after booster immunization. Arthritic severity scores were derived as follows [18]: The clinical scores for paws were classified as 0 (normal joints), 1 (swelling in one digit or joint inflammation), 2 (swelling in two or three digits, or slight paw swelling), 3 (swelling in more than four digits and moderate swelling in the entire paw), and 4 (severe swelling and deformation of the paw). The sum of the scores of all four paws of each mouse represented the total clinical score.

### 2.7. Histology

The left legs and hind paws of mice were removed, fixed with 4% paraformaldehyde in phosphate-buffered saline, and then decalcified for 10 days with EDTA and embedded in paraffin. The paraffin sections were stained with hematoxylin-eosin (HE). The specimens were observed and photographed under a light microscope (DM RAS2 Leica, Solms, Germany). For Safranin O staining, sections were placed in hematoxylin for 2 min and then washed in water for 5 min. Sections were then placed in 0.1% Safranin O solution for a further 2 min and washed again in water for 30 sec. Finally, the sections were passed through a series of industrial methylated spirit concentrations (70% to 100%) for 2 min at each concentration. The sections were then clarified in xylene for 2 min. Sections were then viewed and images taken using a light microscope.

### 2.8. Immunohistochemistry

Immunohistochemical staining for IL-1 was performed. After sections were deparaffinized, rehydrated, and washed, the sections were then antigen-retrieved by pepsin and incubated with 0.3% hydrogen peroxidase for 20 min to block endogenous peroxidase activity, followed by processing with serum for 30 min to block nonspecific ligations. The sections were then treated with rabbit anti-IL-1 (1 : 100, Santa Cruz Biotechnology, CA, USA) primary antibody overnight at 4°C and then washed and incubated with reagents from an immunohistochemical kit (Zhongshan Biotechnology Ltd., Beijing, China) in compliance with the manufacturer's instructions and visualized by 3,3-diaminobenzidine tetrahydrochloride (DAB). Finally, the sections were counterstained with hematoxylin. Sections were then viewed and images taken using a light microscope.

### 2.9. Enzyme-Linked Immunosorbent Assay (ELISA)

Twenty-four hours after the last administration, 0.8 mL of PB was collected from each mouse by eyeball extirpation. Blood samples were incubated at 25°C for 60 min, and sera were isolated by centrifuging at 3000 rpm at 4°C for 10 min. Supernatants were collected and stored at −20°C for protein quantification. The concentrations of IFN*γ*, IL-4, and IL-17 were determined by quantitative sandwich ELISA using ELISA kits (eBioscience, San Diego, CA, USA). According to the general protocol, 100 *μ*L of sample was incubated with 100 *μ*L of biotin labeled antibody for 60 min at 37°C followed by three washes. One hundred microliters of horseradish peroxidase (HRP) labeled avid in fluid was added and incubated for 30 min at 37°C followed by five washes. Then, 90 *μ*L of substrate solution was added and incubated for 25 min at 37°C. Finally, 50 *μ*L of stop solution was added; the optical density (OD) value was read with a Multimode Reader at a wavelength of 450 nm.

### 2.10. Fluorescence Activated Cell Sorter (FACS) Analysis

Spleens were removed, diced, and expressed through a 40 *μ*m Nylon mesh. All isolated spleen cells were made into single-cell suspensions. To quantify the percentage CD3 and CD4 positive cells, cells were washed and stained with anti-mouse-CD3-PE and anti-mouse-CD4-PE-Cyanine5 antibodies (eBioscience). To determine whether the percentage of Th1, Th2, or Th17 cells, cells of all splenocytes and PB were stimulated with phorbol 12-myristate 13-acetate (PMA) (50 ng/mL) and ionomycin (Ion) (1 *μ*g/mL) (Sigma, CA, USA) for 5 h in the presence of GolgiPlug (BD Bioscience, NJ, USA) according to the manufacturer's protocol. Cells were then washed and stained with anti-mouse-CD4-PE-Cyanine5 antibody (eBioscience). Following CD4 staining, cells were blocked, fixed, and permeabilized using a Fixation/Permeabilization kit according to manufacturers' instructions (BD Bioscience, NJ, USA) and then further stained with anti-mouse-IFN*γ*-PE, anti-mouse-IL-4-PE-Cyanine7, or anti-mouse/rat-IL-17A-FITC antibodies. To determine the percentage of Treg cells, cells were washed and stained with anti-mouse-CD4-PE-Cyanine5 and anti-mouse-CD25-PE antibodies. Following CD4 and CD25 staining (eBioscience), cells were blocked, fixed and permeabilized using a Fixation/Permeabilization kit according to manufacturers' instructions (BD Bioscience), and stained with anti-mouse/rat-FOXP3-FITC antibody (eBioscience), Flow cytometry was performed by a FACS Calibur cytometer and analyzed using CellQuest software (Beckman Coulter, CA, USA).

### 2.11. Western Blot Analysis

The spleens of CIA mice were homogenized in 1 mL of a lysis buffer (Sigma, CA, USA). The extracts were clarified by spinning at 10,000 ×g at 4°C for 15 min and then diluted with lysis buffer to achieve a concentration of approximately 2 mg/mL protein. Protein samples were separated by 10% sodium dodecyl sulfate-polyacrylamide gel electrophoresis (SDS-PAGE) and transferred onto nitrocellulose membranes (Amersham Pharmacia Biotech, Uppsala, Sweden). The membranes were incubated with primary antibodies, including rabbit anti-ROR*γ* polyclonal antibody (Abcam, Cambridge, MA, USA), rabbit anti-mouse-T-bet, and anti-mouse-GATA-3 monoclonal antibodies (Santa Cruz, CA, USA), and then incubated with HRP-conjugated secondary antibody (Santa Cruz). All immunoreactive proteins were visualized using SuperSignals West Pico Chemiluminescent Substrate (Thermo Scientific, Rockford, IL, USA). Densitometry plots of the protein expression levels were normalized to GAPDH and expressed relative to the levels in the normal group.

### 2.12. Statistical Analysis

All data are presented as the mean ± standard deviation (SD). Statistical analyses were performed using SPSS13.0 (SPSS Inc., Chicago, IL, USA). One-way analysis of variance (ANOVA) followed by the Tukey-Kramer test for multiple comparisons was used to compare with the treatment groups. A *P* value of < 0.05 was considered statistically significant.

## 3. Results

### 3.1. Characteristics of Pure Compounds in XFHM

**Figure 1:**
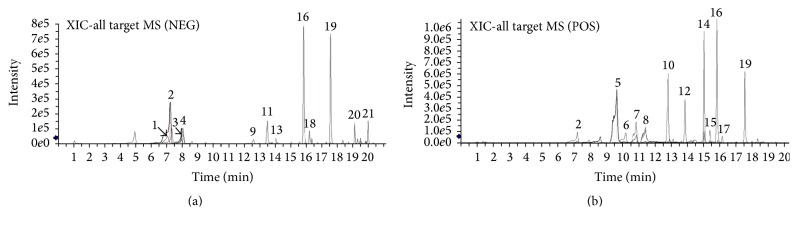
High performance liquid chromatography-electrospray ionization/mass spectrometer ion chromatograms for xianfanghuomingyin. (a) Negative base peak mass spectrometry (MS) spectrum; (b) positive base peak MS spectrum.

In recent years, liquid chromatography-mass spectrometry (LC-MS) has become an essential for the analysis of herbal constituents. It is a powerful analytical tool that has been used to identify compounds in TCM [19–21]. In this study, multistage MS and high resolution MS were performed in both negative and positive ion modes to gain complete information on chemical constitutions of XFHM. The negative base peak MS spectrum and positive base peak MS spectrum are displayed in Figure 1. Twenty-one constituents were identified by the precise mass and relative ion abundance of the target peaks. The identified compounds and biological activities of individual constituents are shown in Table 1. Multiple reports have indicated that these components have biological activities including anti-inflammatory or immune-regulatory functions, inhibitory effects on inflammatory proliferation or angiogenesis, and increased production of anti-inflammatory cytokines. These results provide further evidence to support the therapeutic effects of XFHM in RA treatment.

The toxicity of XFHM in mice was studied in a parallel experiment. The effects on liver and kidney functions are shown in S2 Fig. There were no significant differences between mice in the normal group and mice in the XFHM group.

### 3.2. XFHM Suppresses Histopathological Changes and IL-1*β* Expression in Metatarsophalangeal Joints in CIA Mice

**Figure 2:**
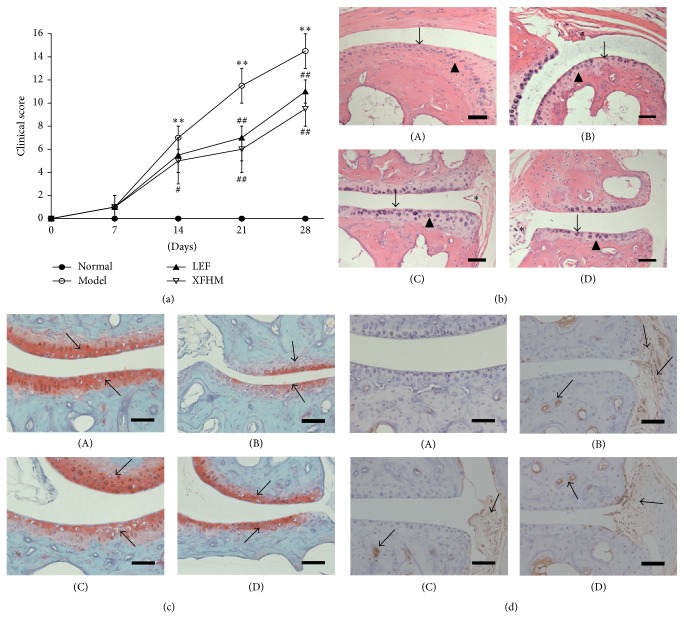
Clinical scores of CIA, hematoxylin-eosin (HE) staining, Safranin O staining, and immunohistochemistry for IL-1*β* in metatarsophalangeal joints. (a) The clinical CIA scores monitored every 7 days after the booster immunization. Data are presented as means ± SD. ^*∗∗*^ *P* < 0.01 compared to the normal group; ^#^ *P* < 0.05, ^##^ *P* < 0.01 compared to the model group. (b) HE staining in metatarsophalangeal joints: ↑ indicates synovium, ▲ indicates articular cartilage, and *∗* indicates pannus. (c) Safranin O staining of the growth plate. The arrows indicate Safranin O staining. (d) Immunohistochemistry for IL-1*β*. The arrows indicate the location of IL-1*β*. Original magnification: 200x; (A) normal group, (B) model group, (C) group treated with LEF, and (D) group treated with XFHM. The scale bar corresponds to 60 *μ*m throughout.

After booster immunization, the clinical CIA scores were determined weekly. As shown in Figure 2(a), on day 14, the swelling score associated with arthritis in the XFHM group was 4.67 ± 1.03 compared to 6.83 ± 0.75 for the normal group. The XFHM and LEF treatments significantly reduced the arthritis severity scores on days 0–28 after booster immunization. Histopathological lesions in the metatarsophalangeal joints of mice were studied after HE staining (Figure 2(b)), and synovial hyperplasia, pannus formation, and destruction of articular cartilage were detected in the metatarsophalangeal joints of CIA mice. Histopathological lesions were alleviated by treatment with LEF and XFHM. Moderate proliferation of synovial cells was observed in XFHM-treated group. Cell morphology was restored to normal. Pannus formation was less and cartilage surfaces were smoother than those of the model group. Bone destruction was prevented by XFHM treatment.

A decrease in Safranin O staining of the growth plates in CIA mice was evident prior to morphological changes in the chondrocytes [22]. There were also differences in Safranin O staining of the growth plates observed among the groups (Figure 2(c)). The intensity of Safranin O staining in CIA mice decreased significantly relative to the normal mice. After being treated with LEF and XFHM, the intensity of staining increased. Immunohistochemical detection and localization showed that IL-1*β* was expressed in the synovium and chondrocytes. As showed in Figure 2(d), significant differences were observed between normal and CIA mice. Abnormal expression of IL-1*β* decreased significantly with LEF and XFHM treatments.

### 3.3. XFHM Inhibits the Proliferation and Differentiation of T Lymphocytes in CIA Mice

**Figure 3:**
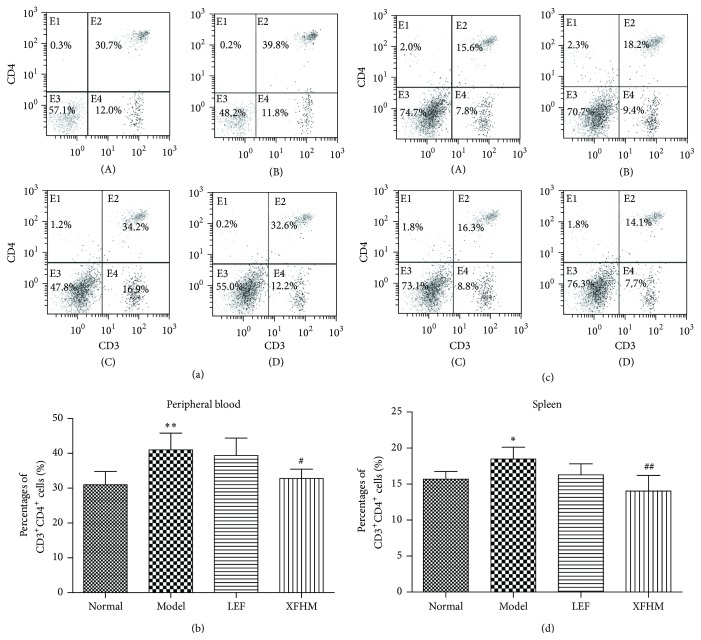
Xianfanghuomingyin attenuated the proliferation of CD3^+^CD4^+^ T cells in peripheral blood and spleen. (a) and (b) The percentage of CD3^+^CD4^+^ T cells in the peripheral bloods of mice after 4 weeks of treatment. (c) and (d) The percentage of CD3^+^CD4^+^ T cells in spleen of mice after 4 weeks of treatment. Results are presented in the bar charts. Data are presented as means ± SD. ^*∗*^ *P* < 0.05, ^*∗∗*^ *P* < 0.01 compared to the normal group; ^#^ *P* < 0.05, ^##^ *P* < 0.01 compared to the model group.

FACS analysis showed that the ratios of CD3^+^CD4^+^ T lymphocytes in PB (Figures 3(a) and 3(b)) and spleen (Figures 3(c) and 3(d)) increased significantly in CIA mice (*P* < 0.01 or *P* < 0.05). These percentages in the treatment groups were lower than those of the model group. T lymphocytes counts in spleen of the XFHM group were decreased significantly relative to those of the model group (*P* < 0.05).

### 3.4. XFHM Decreases the Differentiation of Th1 Cells in Spleen of CIA Mice

**Figure 4:**
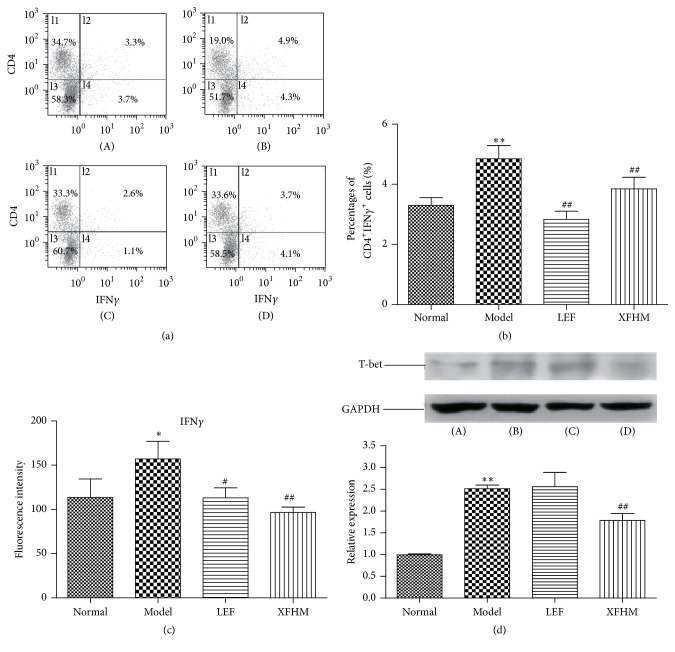
XFHM suppressed the differentiation of CD4^+^IFN*γ* ^+^ Th1 cells and the production of IFN*γ* and T-bet. (a) and (b) The percentage of CD4^+^IFN*γ* ^+^ Th1 cells in spleens isolated from mice treated for 4 weeks. (c) The IFN*γ* levels in sera of mice detected by ELISA. The results are presented in the bar chart. (d) T-bet was detected in whole spleen tissue lysates by western blot analysis. The results are presented in the bar chart. GAPDH was used as an internal control. Data are presented as means ± SD. ^*∗*^ *P* < 0.05, ^*∗∗*^ *P* < 0.01 compared to the normal group; ^#^ *P* < 0.05, ^##^ *P* < 0.01 compared to the model group.

To study the differentiation of Th1 cells, lymphocytes from the spleens of CIA mice were stained with CD4 antibody and, intracellularly, with IFN*γ*, and then a FACS analysis was conducted. As shown in Figure 4, the percentage of Th1 cells in the model group was increased significantly relative to those of the normal group (*P* < 0.01). However, the levels of Th1 cells in the treatment groups decreased significantly relative to those of the model group (*P* < 0.01). Because T-box transcription factor (T-bet) regulates the differentiation of Th1 cells and production of Th1 cytokines, particularly IFN*γ* [23], we tested the levels of IFN*γ* in sera by ELISA, as well as the expression of T-bet in whole spleen tissue lysates by western blot analysis. The IFN*γ* levels in sera and the production of T-bet in spleens of the model group were increased significantly relative to those of the normal group (*P* < 0.01 or *P* < 0.05), but increases in IFN*γ* and T-bet were suppressed with XFHM treatment (*P* < 0.01).

### 3.5. XFHM Increases the Differentiation of Th2 Cells in Spleen of CIA Mice

**Figure 5:**
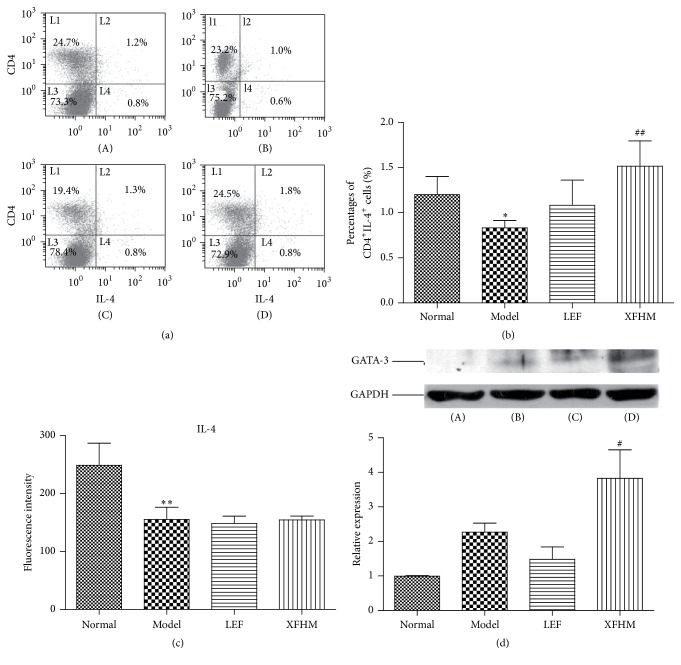
XFHM enhanced CD4^+^IL-4^+^ Th2 cells differentiation and the production of IL-4 and GATA-3. (a) and (b) The percentage of CD4^+^IL-4^+^ Th2 cells in spleen isolated from mice treated for 4 weeks. (c) The level of IL-4 in sera of mice detected by ELISA. The results are presented in the bar chart. (d) GATA-3 was detected in whole spleen tissue lysates by western blot analysis. GAPDH was used as an internal control. Data are presented as means ± SD. ^*∗*^ *P* < 0.05, ^*∗∗*^ *P* < 0.01 compared to the normal group; ^#^ *P* < 0.05, ^##^ *P* < 0.01 compared to the model group.

Whole spleen cells were surface stained by CD4 antibody and intracellularly stained by IL-4 antibody to determine the percentage of Th2 cells. In addition, we examined the transcriptional regulator, GATA-3, that plays a role in Th2 differentiation and cell expansion [24]. We also assayed the IL-4 levels in sera by ELISA and production of GATA-3 in spleens by western blot analysis. As shown in Figure 5, the percentage of CD4^+^IL-4^+^ Th2 cells in spleens and the levels of IL-4 in sera of the model group were less than those of the normal group (*P* < 0.01 or *P* < 0.05). The percentage of Th2 cells and protein expression of GATA-3 in spleens of the XFHM group were higher than those of the model groups (*P* < 0.05).

### 3.6. XFHM Downregulates the Differentiation of Th17 Cells in Spleen of CIA Mice

**Figure 6:**
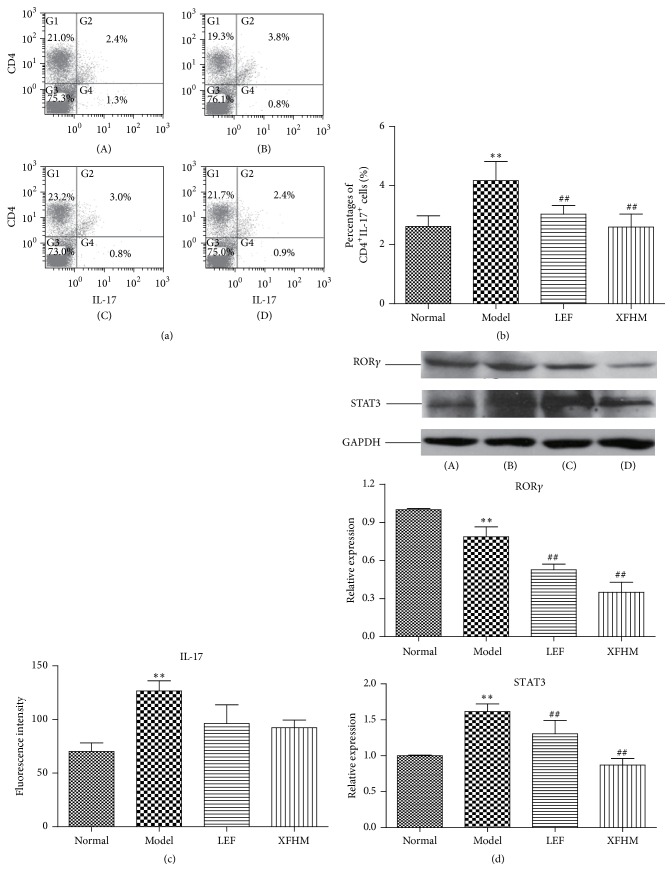
XFHM inhibited CD4^+^IL-17^+^ Th17 cells differentiation and the production of IL-17, STAT3, and ROR*γ*. (a) and (b) The percentage of CD4^+^IL-17^+^ Th17 cells in spleen isolated from mice treated for 4 weeks. (c) The levels of IL-17 in sera of mice detected by ELISA. The results are presented in the bar chart. (d) STAT3 and ROR*γ* were detected in whole spleen tissue lysates by western blot analysis. GAPDH was used as an internal control. Data were presented as means ± SD. ^*∗∗*^ *P* < 0.01 compared to the normal group; ^##^ *P* < 0.01 compared to the model group.

Th17 cell, which exclusively produces IL-17, plays a key pathogenic role in autoimmune diseases. Signal transducer and activator of transcription 3 (STAT3) and RAR-related orphan receptor *γ*t (ROR*γ*t) are nuclear receptors required to generate IL-17-producing CD4^+^ Th17 cells [25, 26]. We stained spleen cells with CD4 and IL-17 antibodies for FACS analysis and measured the levels of IL-17 in mouse sera and the protein expression levels of STAT3 and ROR*γ* in spleens, to explore the biological effects of XFHM on the regulation of Th17 cells differentiations in spleens of CIA mice. Our data showed that the level of Th17 cells in spleens of the model group was markedly higher than that of the normal group (*P* < 0.01), and IL-17 in sera was also increased (Figures 6(a), 6(b), and 6(c)). The percentage of Th17 cells and the production of STAT3 and ROR*γ* in the treatment groups decreased significantly relative to those of the model group, and the effects of XFHM were superior to those of LEF (*P* < 0.01) (Figure 6(d)). The high levels of IL-17 displayed a downwards trend in the XFHM and LEF groups (Figure 6(c)).

### 3.7. XFHM Induces the Differentiation of Treg Cells in PB and Spleen of CIA Mice

**Figure 7:**
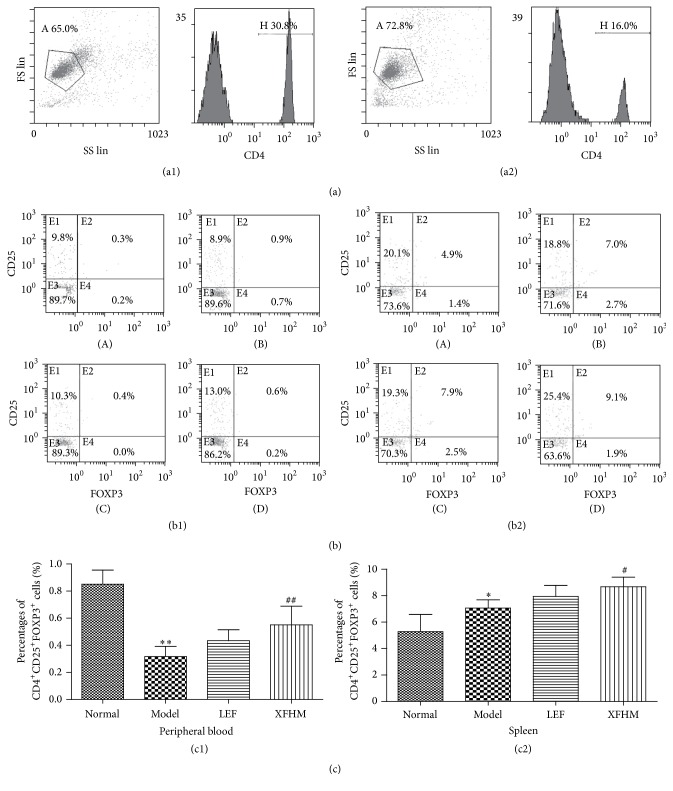
XFHM increased CD4^+^CD25^+^FOXP3^+^ Tregs differentiation. (a) The percentages of CD4^+^ T cells in peripheral bloods (PB) and spleens of mice. Lymphocytes were gated in total cells ((a1) PB, (a2) spleen); (a1) and (a2) are the percentages of CD4^+^ T cells in PB and spleens. (b1) and (b2) are the percentages of CD4^+^CD25^+^FOXP3^+^ Treg cells in PB and spleens of mice after treatment for 4 weeks. (c) The results are presented in the bar chart. Data are presented as means ± SD. ^*∗*^ *P* < 0.05, ^*∗∗*^ *P* < 0.01 compared to the normal group; ^#^ *P* < 0.05, ^##^ *P* < 0.01 compared to the model group.

Treg cells in PB and spleen were stained by CD4 and CD25 antibodies and intracellularly strained by FOXP3 antibody and then evaluated by FACS. As shown in Figure 7, the percentage of Treg cells in PB was lower in the model group than in the normal group (*P* < 0.01), and the numbers of Treg cells in the XFHM and LEF groups were higher than those of the model group. Interestingly, the percentage of Treg cells in the XFHM group was significantly higher than that of model group (*P* < 0.05). These results indicate that XFHM can upregulate differentiation of the Treg subset in CIA mice.

## 4. Discussion

TCM formulae, consisting of plants and minerals, are often prescribed based on clinical experience. Several formulae have been verified as valid complementary or alternative therapies for the treatment of various diseases [27, 28]. In this study, we focused on the Chinese traditional therapy XFHM and its mechanisms of action in improving pathophysiologic characteristics of RA in CIA mice. Negative and positive base peak spectra from XFHM were measured to obtain information on its chemical constituents. As shown in Figure 1 and Table 1, 21 constituents were identified by HPLC-ESI/MS^n^ analysis. The experimental evidences indicate that these constituents, including wanillic acid [29], loganic acid [30], paeoniflorin [31], loganin [32], apiin [33], gentiopicroside [34], astragaloside IV [35], acteoside [36], prime-O-glucosylcimifugin [37], and isoimperatorin [38], had anti-inflammatory and immune-regulatory effects that inhibited the activation of NF-*κ*B or other signaling pathways. Chlorogenic acid [39], caffeic acid [40], and wogonin [41] had inhibitory effects on proliferation of synoviocytes and damage to articular chondrocytes. In addition, wogonoside inhibits LPS-induced angiogenesis both* in vitro* and* in vivo* [42]. Naringenin can manipulate the immunostimulatory properties of dendritic cells [43], and formononetin enhances IL-4 production in T cells [44]. Therefore, we suggest that the functions of the Chinese herbs in XFHM contribute to its actions on multiple targets and its synergistic therapeutic effects against RA.

RA is an autoimmune rheumatic inflammatory disease. T cells have been shown to participate in the pathogenesis of RA. For many years, RA has been considered as Th1-dependent disease. Autoreactive T cells may recognize a number of autoantigens, induce Th1 cell differentiation, and disturb the balance of Th1 and Th2 cells, triggering the autoimmune response that contributes to the pathogenesis of RA [45]. This concept has changed since the discovery of Th17 cells. Th17 cells, characterized by production of the highly inflammatory cytokine IL-17, mediate joint pathology. These cells, which also produce INF*γ* and other inflammatory cytokines, initiate and prolong synovitis in multiple joints and facilitate pannus development, which eventually lead to cartilage and bone destruction [46, 47]. In addition, deficiencies in the number and function of Treg cells and resistance of effector T cells to Treg cell mediated suppression are involved in the development of synovial inflammation [7]. CIA mice are a recognized animal model for RA research. The proliferation and differentiation of T cells in CIA mice were reflected in pathological changes similar to RA [48].

In the pathogenesis of RA, antigen-presenting cell provides immune cues that instruct the differentiation of naïve CD4^+^ precursor cells to optimally counteract infectious threat [49]. Separate lineages of effector Th-cell differentiated from naïve CD4^+^ precursor cells participate in immunological response. The Th1 cell lineage is characterized by production of IFN*γ*, whereas the Th2 cell lineage is characterized by production of IL4, IL5, and IL13 [50]. Differentiation of Th1 and Th2 effector cells is governed by the cytokines IL-12 and IL-4, as well as by signaling through the Notch receptor [51]. Binding of the initial auto/paracrine produced IFN*γ* to its receptor activates STAT1 and then strongly promotes the expression of the Tbx21 gene, which encodes T-bet. T-bet enhances the transcriptional competence and increases production of INF*γ*. Furthermore, T-bet increases expression of the IL-12 receptor *β*2 chain and further enhances IFN*γ* production. T-bet also prevents Th2 differentiation by inhibiting expression of the GATA-3 gene [52]. For the differentiation of Th2 cells, IL-4 receptor signaling strongly promotes expression of the IL-4 and GATA-3 genes. GATA-3 encompasses the IL-4, IL-5, and IL-13 genes and promotes production of these cytokines. Finally, IL-4 enhances Th2-cell differentiation in a feed-forward loop. GATA-3 can also inhibit the expression of the IL-12 receptor *β*2 chain to restrict Th1 differentiation [53]. Expression of the Tbx21 gene can increase expression of T-bet, leading to transactivation of the IFN*γ* gene by binding to NF-*κ*B p50 or inhibiting of IL-4 receptor signaling [54]. Many reports have suggested that IL-17 and Th17 cells play important roles in induction and propagation of autoimmunity in animal models, as well as human autoimmune disease, including RA [55]. Th17 cells that are specific for self-antigens initiate inflammation and cause severe autoimmunity. The activation of naïve T cells induced by proinflammatory cytokines initiates differentiation of Th17 cells, leading to the expression of transcription factor ROR-*γ*t and production of IL-17 [56]. Our results show that XFHM can inhibit the proliferation and activation of CD3^+^CD4^+^ T cells and, conversely, promote the conversion of T cells into CD4^+^CD25^+^FOXP3^+^ Treg cells. However, XFHM treatment does not regulate the production of granulocyte-macrophage colony-stimulating factor (GM-CSF), which is a critical cytokine for the differentiation of Treg cells (S3 Fig.). Furthermore, XFHM can also suppress the differentiations of Th1 and Th17 cells and increase the differentiation of Th2 cells. According to these results, we suggest that XFHM maintains immunological tolerance by regulating the differentiation of T cell subsets to restore the balance between T lymphocytes. To illustrate the regulatory mechanisms of XFHM, the production of defining proinflammatory cytokines and transcription factors related to Th cells was evaluated. Interestingly, XFHM decreased the levels of IFN*γ*, T-bet (Th1), IL-17, STAT3, and ROR*γ* (Th17) but increased the production of IL-4 and GATA-3 (Th2) to regulate the differentiations of Th subsets.

LEF is a low molecular weight isoxazole derivative used in RA therapies. It has functions for anti-inflammatory and immunosuppressive function [57]. The active metabolite of LEF can prevent the interaction of T cells with antigen-presenting cells that forms an immunologic synapse, regulate the proliferation and differentiation of Th cells to maintain the immunological tolerance, and relieve RA immune-disorders [58]. However, a recent study suggests that LEF has myelosuppressive and hepatotoxic potential, based on a rat model of RA [59]. Adverse drug reactions to LEF, such as pruritus, loss of appetite, weakness, dizziness, diarrhea, and erythema, also occur frequently in clinical follow-up survey. XFHM consists of 12 types of medicinal herbs. The prescription is determined based on clinical symptoms and the compatibility of herbs. Multiple components can act on various targets and have synergistic therapeutic effects. Further, use of XFHM as an alternative therapy for RA can allow patients to avoid myelosuppressive, hepatotoxic, and other potential adverse reactions of modern pharmacotherapies including LEF treatment. In addition, our study also indicates that the therapeutic effects of XFHM are equal to or exceed those of LEF.

In conclusion, our data suggest that XFHM can modulate the differentiations of Th1, Th2, and Th17 cells and promote the differentiation of Treg cells, restoring the normal balance of Ths cells and maintaining immunological tolerance, interfere with immune cell invasion of synovial membranes, and prevent cartilage and bone destruction in joints of RA joints. XFHM, therefore, should be used as a complementary or alternative traditional medicine in the treatment of RA.

## Supplementary Material

S1 Fig. The infrared spectrum fingerprint (IRFP) of XFHM compound.
S2 Fig. Effect of XFHM compound on BUN, Cr, ALT and AST.
S3 Fig. Effect of XFHM compound on GM-SCF in sera.

## Figures and Tables

**Table 1:** Chemical components identified in xianfanghuomingyin by high performance liquid chromatography-electrospray ionization/mass.

Number	Fragments (*m*/*z*)	Formula	Identification	Biological activities
1	/152.0, 132.0	C_8_H_8_O_4_	Vanillic acid	Anti-inflammatory & immune-regulatory
2	321.1, 169.1	C_16_H_24_O_10_	Loganic acid	Anti-inflammatory & immune-regulatory
3	/135.0	C_9_H_8_O_4_	Caffeic acid	Inhibition on inflammatory proliferation
4	/173.0, 135.0	C_16_H_18_O_9_	Chlorogenic acid	Inhibition on inflammatory proliferation
5	195.1, 121.1	C_16_H_20_O_9_	Gentiopicroside	Anti-inflammatory & immune-regulatory
6	179.1, 151.1	C_23_H_28_O_11_	Paeoniflorin	Anti-inflammatory & immune-regulatory
7	179.1, 149.0	C_17_H_26_O_10_	Loganin	Anti-inflammatory & immune-regulatory
8	77.0, 105.1	C_10_H_10_O_3_	Methyl 4-hydroxycinnamate	Uncertain in autoimmune disease
9	/383.1, 473.1	C_26_H_28_O_14_	Apiin	Anti-inflammatory & immune-regulatory
10	307.1, 261.1	C_22_H_28_O_11_	Prim-O-glucosylcimifugin	Anti-inflammatory & immune-regulatory
11	/491.1, 271.0	C_30_H_32_O_15_	Galloylpaeoniflorin	Uncertain in autoimmune disease
12	259.1, 235.1	C_16_H_18_O_6_	Cimifugin	Uncertain in autoimmune disease
13	/461.2, 161.0	C_29_H_36_O_15_	Acteoside	Anti-inflammatory & immune-regulatory
14	267.07 252.04	C_22_H_22_O_9_	Ononin	Uncertainty in autoimmune disease
15	301.1, 167.1	C_23_H_26_O_10_	Lactiflorin	Uncertainty in autoimmune disease
16	220.1, 225.1 /268.0, 211.0	C_16_H_12_O_5_	Wogonin	Inhibition of inflammatory cell proliferation
17	270.1, 285.1	C_22_H_20_O_11_	Wogonoside	Inhibition for angiogenesis
18	/151.0, 119.1	C_15_H_12_O_5_	Naringenin	Immunostimulatory for dendritic cells
19	197.1, 226.1 /252.0, 223.0	C_16_H_12_O_4_	Formononetin	Increased IL-4 production
20	/254.1, 210.1	C_16_H_14_O_4_	Isoimperatorin	Anti-inflammatory & immune-regulatory
21	/621.40, 651.41	C_41_H_68_O_14_	Astragaloside IV	Anti-inflammatory & immune-regulatory
