# Supplementary material for: Xianfanghuomingyin, a Chinese Compound Medicine, Modulates the Proliferation and Differentiation of T Lymphocyte in a Collagen-Induced Arthritis Mouse Model
Source: Evid Based Complement Alternat Med. 2016 Aug 30;2016:6356871. doi: 10.1155/2016/6356871 (PMC5021507; doi:10.1155/2016/6356871)
Supplement: Supplementary file 1 — S1 Fig. The infrared spectrum fingerprint (IRFP) of XFHM compound. S2 Fig. Effect of XFHM compound on BUN, Cr, ALT and AST. S3 Fig. Effect of XFHM compound on GM-SCF in sera. [file 6356871.f1.docx]

**Xianfanghuomingyin, a Chinese Compound Medicine, Modulates the Proliferation and Differentiation of T Lymphocyte in** **Collagen-Induced Arthritis Mice**

**Infrared fingerprint spectrum (IRFP) techniques for Quality control of Xianfanghuomingyin**

As shown in S1 Fig, the strongest peaks in the range of 860.96-1243.32 cm-1 mainly attributed to the stretching vibration of C-O, which displayed the characteristic absorption of glycosides and terpene lactones, the stronger peak at 3339.14 cm-1 belonging to stretching vibration of O-H groups in associated phenolic hydroxyl of flavonoids and phenol acids, the peaks at 2933.83 and 1414.40 cm-1 assigning to the stretching vibration of –CH2 and –CH3 groups corresponding to alkanes and alkenes, and the peaks in the range of 707.82-439.24 cm-1 attributed to the stretching vibration of C-X pertained to the characteristic absorptions of chlorides and bromides.


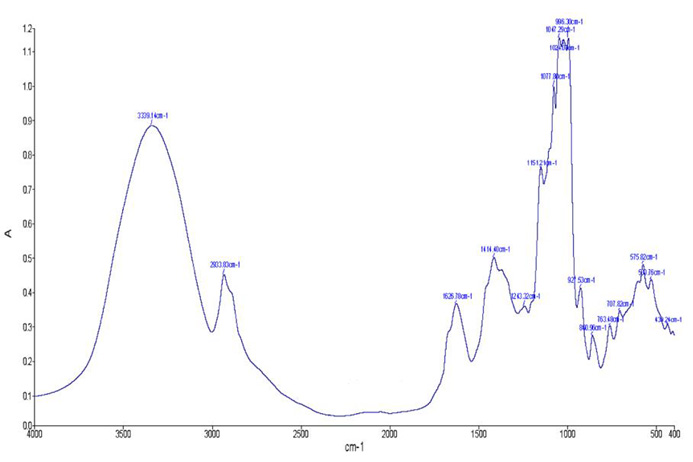


**S1 Fig. The infrared spectrum fingerprint (IRFP) of XFHM compound.** The x-axis indicated wavelength of absorption, and y-axis indicated absorption intensity.

**Effects of XFHM on hepatic and renal functions of CIA mice**

As shown in S2 Fig, there were no significant differences on BUN, Cr, ALT and AST in CIA mice sera of four groups. This result confirmed that XFHM have no damage on hepatic and renal functions of CIA mice.


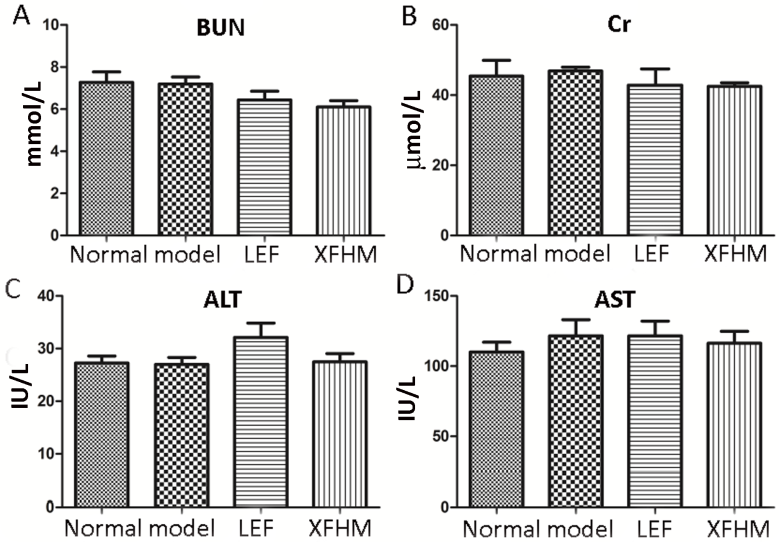


**S2 Fig. Effect of XFHM compound on BUN, Cr, ALT and AST.** A BUN, B Cr, C ALT, D AST.

**Effect of XFHM compound on granulocyte-macrophage colony-stimulating factor (GM-CSF) in sera of CIA mice**

The data of ELISA showed that there were no significant differences on the level of GM-CSF in sera among these groups (S3 Fig). This result indicated that XFHM treatment has no regulatory function on the production of GM-CSF, which is a critical cytokine for the differentiation of Treg cells.


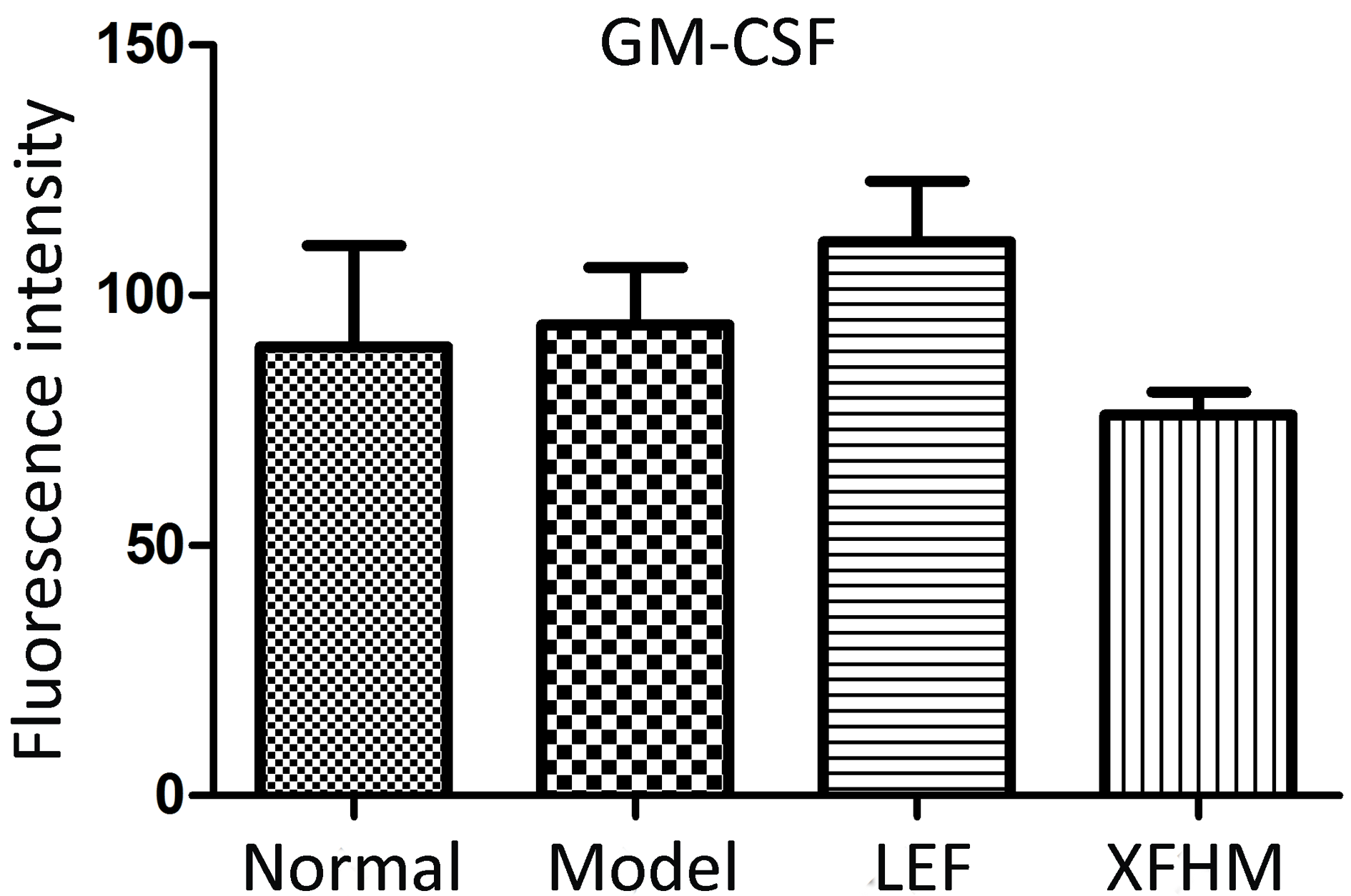


**S3 Fig. E****ffect of XFHM compound on GM-SCF in sera.**
